# Fitness and neural complexity of animats exposed to environmental change

**DOI:** 10.1186/1471-2202-16-S1-P262

**Published:** 2015-12-18

**Authors:** Larissa Albantakis, Giulio Tononi

**Affiliations:** 1 Department of Psychiatry, University of Wisconsin, Madison, WI, 53719, USA

## 

We recently showed that adaptive logic-gate networks ('animats') increase their capacity to integrate information as they adapt to environments of increasing complexity [1]. The animats' task environments consisted of falling blocks of different sizes, which had to be either caught or avoided. When the animats were exposed to more difficult task environments that required more internal memory, they developed more complex networks ('brains'), as indicated by a larger number of irreducible internal mechanisms ('concepts') and higher integrated conceptual information (*Φ*) [2,3]. Animats with brains of high *Φ *outperformed animats with modular or feedforward brains because they could pack a larger number of mechanisms for the same number of nodes and connections. Here we investigate whether this key feature of animats of high *Φ *leads to greater flexibility in adapting to environmental changes. We selected animats with integrated (*Φ *> 0) or modular (*Φ *= 0) structures that had adapted perfectly, within 30,000 generations, to a particular task environment. We then enhanced the difficulty of the task environment by adding blocks of various sizes that were to be caught or avoided in a way that was either: i) congruent with the old task, ii) neutral to the old task, or iii) incongruent with the old task, reversing the previous rules. For congruent changes in the task environment, the fitness of animats with integrated brains and many irreducible mechanisms, as compared to modular animats, (1) dropped less right after the change, (2) recovered faster, and (3) maintained higher values even after 30,000 generations in the new environment. These results corroborate the hypothesis that brains with high capacity for information integration may provide an evolutionary advantage in complex, changing environments.
